# Evaluation of Immunohistochemical Biomarkers in Diabetic Wistar Rats with Periodontal Disease

**DOI:** 10.3390/jpm14050527

**Published:** 2024-05-15

**Authors:** Ioana Scrobota, Ioan Andrei Tig, Andrea Olivia Marcu, Georgiana Ioana Potra Cicalau, Liliana Sachelarie, Gilda Iova

**Affiliations:** 1 Department of Dental Medicine, Faculty of Medicine and Pharmacy, University of Oradea, 1st Decembrie Street, 410073 Oradea, Romania; ioana_scrobota@uoradea.ro (I.S.); itig@uoradea.ro (I.A.T.); cicalau.georgiana@uoradea.ro (G.I.P.C.); gilda_iova@uoradea.ro (G.I.); 2 Preclinics Department, Faculty of Medicine and Pharmacy, University of Oradea, 410073 Oradea, Romania; omarcu@uoradea.ro; 3 Preclinics Department, Faculty of Medicine, Apollonia University, 700511 Iasi, Romania

**Keywords:** dentistry, periodontology, periodontal disease, diabetes, CD3, CD20, CD34

## Abstract

Background: The association of periodontal disease and diabetes is a subject of intense research in terms of etiopathology and treatment options. This research aimed to evaluate the modulation of the local inflammatory status by two natural extracts, curcumin (Cu) and rutin (R), in an experimentally induced diabetes and periodontal disease in Wistar rats. Methods: Fifty Wistar albino rats were randomly assigned to five groups: Control (C), Diabetes-associated Periodontal Disease (DP), Diabetes-associated Periodontal Disease treated with Curcumin (DPCu), Diabetes-associated Periodontal Disease treated with Rutin (DPR), and Diabetes-associated Periodontal Disease treated with both Curcumin and Rutin (DPCuR). Gingival samples were collected from all rats, and immunohistochemical markers CD3, CD20, and CD34 were evaluated to assess the local inflammatory infiltrate. Descriptive statistics were applied (SPSS24 Software, Armonk, NY, USA). Results: Rutin, alone or combined with Curcumin, reduced CD3-positive cell levels. Curcumin demonstrated superior efficacy in reducing CD20-positive cells. The combination of Curcumin and Rutin had the most important impact on both markers. Curcumin notably increased immature CD34-positive cell levels. Conclusions: Curcumin and Rutin, either alone or together, hold potential for reducing local inflammation in diabetes-induced periodontal disease in Wistar rats.

## 1. Introduction

One of the primary contributors to adult tooth loss is periodontal disease [1], while diabetes is associated with a diminished quality of life, reduced life expectancy, increased morbidity from microvascular issues, and an elevated risk of macrovascular complications [2,3]. Notably, periodontal diseases are more likely to manifest in individuals with diabetes, but conversely, uncontrolled diabetes is also linked to a heightened risk of both the onset and progression of periodontal disease [4,5].

The inflammatory response plays a pivotal role in the development of both periodontal disease and diabetes [6,7]. Periodontal disease, stemming from microbial infections, is intricately entwined with the host’s immune system, which can mitigate tissue damage either by eradicating pathogens or by neutralizing their products. This immune response significantly contributes to the destruction of both hard and soft tissues, underscoring its dual function as a protective and destructive mechanism [8]. T and B lymphocytes are believed to be key players in the etiology of periodontal disease, and they are found in both healthy and affected periodontal tissues [9].

In the initial stage of PD, an acute injury under the form of exudative vasculitis in the gingiva takes place. polymorphonuclear leukocytes’ (PMN) migration and macrophages accumulation are a part of the immune response of the organisms to pathogens. In the continuous presence of microbial biofilm, inflammation may not be possible to be limited at this stage. Microorganisms trigger the antigen-presenting cells and a gingival dense infiltration of mononuclear cells, predominantly T lymphocytes, is formed. The T cells’ immune response consists mainly in increasing phagocytosis, but, if it is overwhelmed, a B lymphocyte’s response is activated and predominates with antibodies being produced as a subsequent control of infection. Continuous accumulation of B lymphocytes and release of nonprotective antibodies contribute to PD progression when the alveolar bone and the periodontal ligaments are interested [10,11,12,13].

In response to the injury, all immune cells in contact with pathogens produce cytokines, metalloproteinases, prostaglandins, and proteolytic enzymes. Interleukin (IL)-1β, IL-6, IL-8, IL-12, IL-17, and tumor necrosis factor (TNF)-α [G] were found to be responsible for tissue alterations. Matrix metalloproteinases (MMPs) and prostaglandins, followed by receptor activator of nuclear factor kappa B ligand (RANKL), are responsible for bone resorption [14,15,16].

The inflammation in the periodontal disease and the direction in which it develops depend both on the local microbial status and on the host’s immune response to it [7,10].

Recent research highlights the exacerbation of periodontal diseases due to immune cell alterations in individuals with diabetes [17].

Hyperglycemia in diabetes produces important microvascular and macrovascular alterations resulting in inflammation and decreased healing capacity. PMN and macrophages are the first immune cells that respond. And both inflammatory mediators IL-1β, IL-6, IL-8, IL-17, TNF-α, and anti-inflammatory mediators TGF-β, IL-4, IL-10 are secreted. This immune response, by itself, has an impact on the periodontal tissues. The association of diabetes with periodontal disease creates the most appropriate conditions for the progression of periodontal tissue alterations [18].

When diabetes is not controlled, advanced glycation end-products (AGEs) are accumulating and interact with RAGE pattern recognition receptors on the surface of macrophages and fibroblasts and activate Nuclear factor kappa B (NF-κB) and p38 mitogen-activated protein kinases (p38 MAPK), important pathways in inflammation [19,20].

Given that the immune-inflammatory response is the primary driver behind periodontal tissue destruction, there is growing interest in host-modulating treatment approaches utilizing natural therapeutic agents. Practically, any agent that can modulate the mechanisms implicated in periodontitis and diabetes could be considered as a potential candidate [21].

Curcumin (Cu) boasts a plethora of biological properties, including anti-inflammatory, antioxidant, antimicrobial, and antiviral effects. These qualities position Cu as a promising avenue for the treatment of periodontal disease [21,22].

In vitro and in vivo studies attest that the anti-inflammatory effect of curcumin is due to a reduction of the cellular immune response to the microbial biofilm leading to a prevention in periodontal tissue destruction [23]. In diabetes associated with periodontitis, a modified form of Cu seems to have anti-inflammatory effects and improvement of diabetic osteoporosis, even in maintained hyperglycemic conditions [24]. Administered locally curcumin showed a reduction of the oxidative stress biomarker, malondialdehyde, and antioxidant enzymes amending periodontitis in diabetic rats [25]. Periodontal pathogenic microorganisms were inhibited by topically applied Cu. While demonstrating good biocompatibility, Cu hydrogels successfully prevented the loss of alveolar bone. [26].

*P. gingivalis*, *Prevotella intermedia*, *Fusobacterium nucleatum*, *Treponema denticola*, and periodontal pathogens were inhibited by curcumin [27]. Compared to chlorhexidine, a commonly used periodontal antiseptic, curcumin showed similar effects on *A. actinomycetemcomitans*, *P. gingivalis*, and *Prevotella intermedia* [28].

Rutin (R), a flavonoid found in various plants, exhibits a wide array of biological activities, encompassing antidiabetic, antioxidant, and anti-inflammatory properties. This versatility makes R an attractive candidate for addressing complications associated with diabetes [29,30]. A recent double-blind, placebo-controlled trial reported rutin to have normalized glycemia, increased insulin sensitivity, and improved the lipidic profile and the oxidative stress status as well as reduced inflammation in patients diagnosed with diabetes [31]. In an in vitro study, rutin modulated the oxidative stress by reducing the production of reactive oxygen species and augmenting the antioxidant activity. R stimulated the proliferation of periodontal ligament stem cells in the absence of any treatment [28]. We found little evidence of the effect of R in periodontitis associated with diabetes. In previous research, we found that rutin, alone or in combination with curcumin, exhibited general and local antioxidative properties in periodontitis associated with diabetes [30].

The health of periodontal tissue, a highly vascularized structure, is paramount for its proper function. Some research has delved into gingival vascular changes in diabetic patients with periodontal disease, although limited data is available on this topic [32,33].

The overarching goal of this study was to assess the inflammatory infiltrates within gingival tissues obtained from Wistar rats afflicted with periodontal disease compounded by diabetes. Furthermore, we aimed to investigate how this inflammatory response is modulated while delivering Cu, R, and both extracts together, in that order.

## 2. Materials and Methods

### 2.1. Substances

The following substances were acquired from Sigma-Aldrich^®^ Chemie GmbH, Munich, Germany: streptozotocin, glucose, curcumin (yellow to orange powder, soluble in EtOH (10 mg/mL) purity (HPLC) > 65%, CAS Number: 458-37-7), rutin (yellow powder, soluble in Pyridine (50 mg/mL), purity (HPLC) > 94%, CAS Number: 207671-50-9), 2-thiobarbituric acid, o-phthalaldehyde, hydrogen peroxide ACS reagent and kalium phosphate buffer. Mineral oil (S.C. Vitamar Import Export SRL, Bucharest, Romania) was used to disperse Cu and R at a concentration of 10 µmol/L, while distilled water (S.C. Vitamar Import Export SRL, Bucharest, Romania) was used to dissolve streptozotocin and glucose. Immunohistochemistry markers CD3 (Dako, clone F7.2.38), CD20 (Dako, clone L26), and CD34 (Dako, clone QBEnd10) were procured from Dako Corp (Glostrup, Denmark).

### 2.2. Animals

Fifty Wistar albino rats, all male, 8 weeks old, weighing 220 ± 20 g on average, were obtained from the “Iuliu Hațieganu” University of Medicine and Pharmacy in Cluj-Napoca, Romania’s Animal Department. After that, the rats were moved to and housed in the Department of Physics’ BIOCOM Research Centre. They were kept in a controlled environment with a temperature of 21 ± 2 °C, a humidity of 70 ± 4%, and a 12-h light/12-h dark cycle for the duration of the study. The rats were kept in cages with five rats apiece, fed a typical laboratory diet of pellets, and allowed unlimited access to water [34].

### 2.3. Diabetes Mellitus and Periodontal Disease Induction

The rats were randomly allocated into five groups as follows: Group 1 served as the control group (C); Group 2 represented the diabetes-associated periodontal disease group (DP); Group 3 consisted of rats with periodontal disease associated with diabetes and treated with Cu (DPCu); Group 4 included rats with periodontal disease associated with diabetes and treated with R (DPR); Group 5 comprised rats with periodontal disease associated with diabetes and treated with a combination of Cu and R (DPCuR).

Rats in groups 2 through 5 were given an intramuscular anesthetic with a ketamine-xylazine cocktail (100 mg/kg ketamine and 10 mg/kg xylazine) after they had been acclimated for one week. Their intravenous dosage of streptozotocin (30 mg/kg body weight) was then given to them, and six hours later, each animal was given 2 mL of 30% glucose to cause diabetes. The identical volume of a vehicle solution was the sole thing provided to rats in Group 1. Rats whose glycemia was less than 300 mg/dL were subjected to another round of the streptozotocin and glucose induction regimen after their blood glucose levels had been tested two days later using a glucometer [30,34]. Around the second mandibular rat molars, 0.1-mm-diameter stainless steel ligature wires were positioned in groups 2 to 5. Ligatures were monitored three times a week and, if necessary, substituted. The periodontal disease was installed after 15 weeks when an average pocket depth of 3.1 mm was measured in the ligatured teeth [30,34].

### 2.4. Treatment Protocol

Following the establishment of diabetes and periodontal disease, rats in groups DPCu, DPR, and DPCuR received daily oral gavage treatments. These treatments included Cu (75 mg/kg body weight per rat), R (75 mg/kg body weight per rat), and an equal mixture of Cu and R (75 mg/kg body weight per rat), respectively [31]. As previously reported [31], the doses were established from scientific articles regarding the effects of Cu and R on inflammatory parameters. The values we found varied between 15–90 mg/kg for C and 25–100 mg/kg for R.

After ten weeks of treatment, at the conclusion of the experiment, the animals were anesthetized via intraperitoneal injection of a ketamine-xylazine cocktail (90 mg/kg body weight of ketamine and 10 mg/kg body weight of xylazine). Tissue samples were then collected from the gingival mucosa of each animal for subsequent immunohistochemical analysis [34].

### 2.5. Immunohistochemistry

T and B lymphocytes were evaluated in gingival tissue samples collected from the research animals. This assessment involved the use of a cluster of differentiation 3 (CD3), a protein complex and T cell co-receptor [35,36], and a cluster of differentiation 20 (CD20), a protein expressed on the surface of B cells [37]. We closely observed alterations that occurred within the gingival epithelium, as well as in the superficial and deep chorion layers. Special attention was given to changes in blood vessels, along with an examination of the inflammatory infiltrates within the chorion. To quantify endothelial cells in the gingival connective tissue, we utilized the immunohistochemistry vascular marker cluster of differentiation 34 (CD34), which selectively labels both endothelial cells and angioblasts (Table 1). This CD34 antibody was employed in our immunohistochemistry investigation to elucidate vascular modifications within the periodontium [38].

Following standard protocol, tissue specimens were embedded in paraffin after being preserved for up to 24 h in 10% buffered formalin (pH 7.4). Each paraffin-embedded tissue was sectioned at 4 μm using a Leica RM 2125 R T with a thin profile Dura Edge type microtome (Leica Biosystems, Deer Park, IL, USA), in accordance with a standard automated immuno-histochemical protocol (Ventana GX autostainer; Ventana Medical System, Tucson, AZ, USA) [39]. Immunohistochemistry was used to detect CD3 (Dako, clone F7.2.38), CD20 (Dako, clone L26), and CD34 (Dako, clone QBEnd10). CD34+ blood vessels that showed lumen were considered mature vascular structures, and immature blood vessels were considered those CD34+ vascular structures that did not show vascular lumen or CD34+ progenitor cells [40]. The identical protocol was applied to the negative controls, with the exception of the primary antibody. For each biomarker, normal palatine tonsils served as the positive control. The palatine tonsil is mainly a lymphoid organ. Since the immune response was studied, in our study, by evaluating B lymphocytes (CD20) and T lymphocytes (CD3), this organ is recommended to be used as a positive control [41]. Being intensively vascularized, like any internal organ, the palatine tonsil is recommended as a positive control for the CD34 marker (vascular marker) [42,43]. All of the evaluation was completed using the hot-spot method. Three standard fields of 0.5/0.5 cm were selected for each case from areas with high densities of the investigated antibody, and the number of positive cells from 100 cells was tallied. The outcomes and the features of normal tissue were connected.

### 2.6. Statistical Analysis

The statistical analysis was performed in the SPSS24 Software (version 24, Armonk, NY, USA) dedicated to statistical processing. A level of significance of 0.05 was considered. Data were processed with descriptive statistics. For assessing the significance of differences between groups we used an inferential statistic, a *t*-test [34].

## 3. Results

The number of CD3+ T cells increased, in average, in the group of rats with diabetes associated to periodontal disease (DP) as compared to the control group (Table 2).

There is a difference considered to be extremely statistically significant (*p* ˂ 0.0001) between CD3+T, CD20+ B in all groups, Table 3.

**Figure 1:**
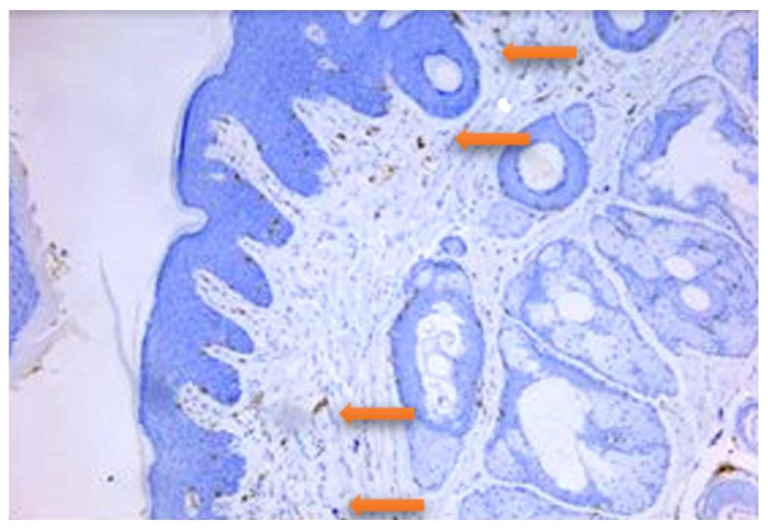
Rat, gingiva. CD3 immunolabelled type T lymphocytes are seen in the submucosa in a band like distribution (some indicated by the arrows); 100×.

The distribution of CD3+T lymphocytes was diffuse and inhomogeneous, very rarely being identified grouped, perivascular, or subepithelial (Figure 1).

**Figure 2:**
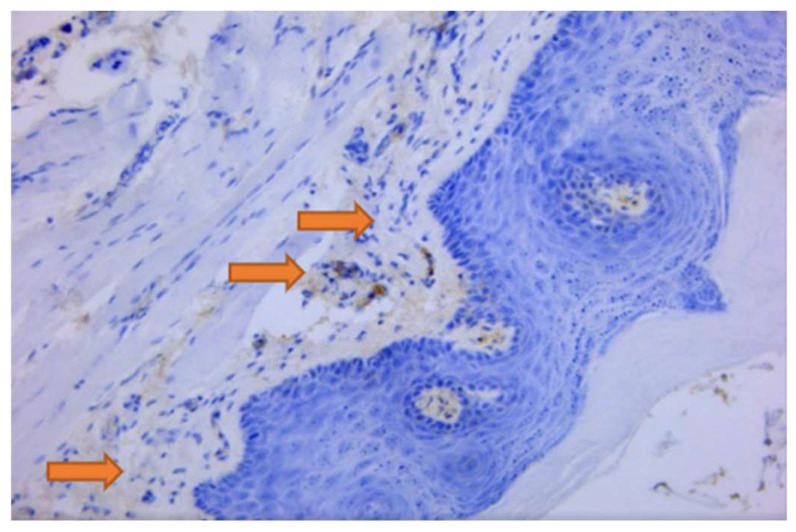
Rat, gingiva. Multiple CD3 immunolabelled type T lymphocytes with a predominatly perivascular location; 200×.

In the DP group compared to the control group, a larger proportion of CD3+ T lymphocytes than CD20+ B lymphocytes were found; Table 3 (*p* ˂ 0.0001). CD3+T lymphocytes were the most prevalent immune system cells, according to the immunohistochemical examination of inflammatory infiltrate cells in the gingival tissue of DP Wistar rats. They were irregularly distributed throughout the periodontium’s connective tissue, with the majority of CD3+ T lymphocytes found at the level of the covering and perivascular epithelium (Figure 2).

**Figure 3:**
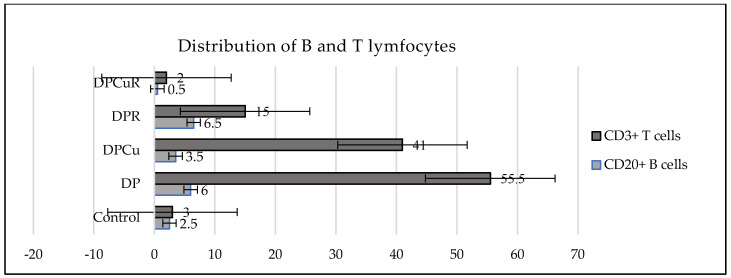
Mean values of B and T lymphocytes in the gingival inflammatory infiltrate of all studied groups: Control-control group; DP-diabetes associated with periodontal disease group; DPCu-diabetes associated with periodontal disease treated with curcumin group; DPR-diabetes associated with periodontal disease treated with rutin group; DPCuR-diabetes associated with periodontal disease treated with curcumin and rutin group. Significant differences (*p* < 0.05) were depicted between CD3+ T cells and CD20+ B cells within each group.

In contrast to CD3+T lymphocytes, B lymphocytes were less represented in the inflammatory infiltrates of periodontal tissue in all groups compared to controls and were found perivascular or at the chorion level. The group of diabetic rats with periodontal disease treated with the combination of Cu and R (DPCuR) showed an important reduced number of B lymphocytes compared to the DP group and the groups treated with Cu or R alone (Figure 3).

**Figure 4:**
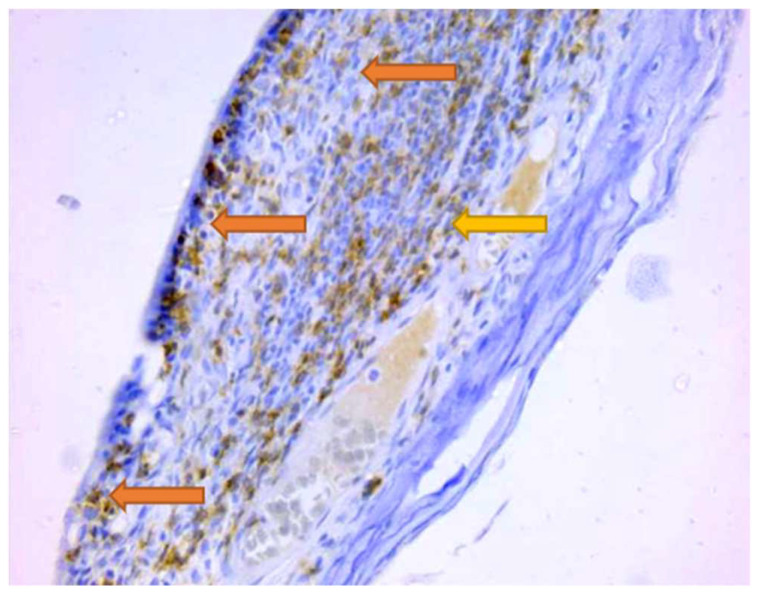
Rat, gingiva. Many CD20 immunolabelled type B lymphocytes are seen in the submucosa with a multifocal to nodular distribution; 200×.

On some sections, CD20+ B lymphocytes were the most prevalent cells in the inflammatory areas. They were either seen to have a diffuse appearance, one concentrated around the blood vessels, or a nodular appearance (Figure 4).

**Figure 5:**
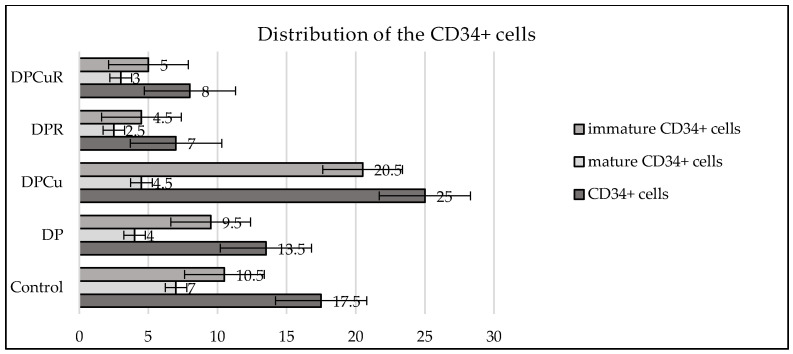
Mean values of CD34+ cells in the inflammatory infiltrate of all studied groups: Control—control group; DP—diabetes associated with periodontal disease group; DPCu—diabetes associated with periodontal disease treated with curcumin group; DPR—diabetes associated with periodontal disease treated with rutin group; DPCuR—diabetes associated with periodontal disease treated with curcumin and rutin group.

The overall number of CD34+ cells decreased in the DP group compared to the control group. Also, the total number of progenitor cells decreased when R was administered as monotherapy or in combination with Cu. In DP rats, R, followed by Cu combined with R decreased the number of circulating immature progenitor cells. Cu administered alone increased the number of immature progenitor cells (Figure 5).

**Figure 6:**
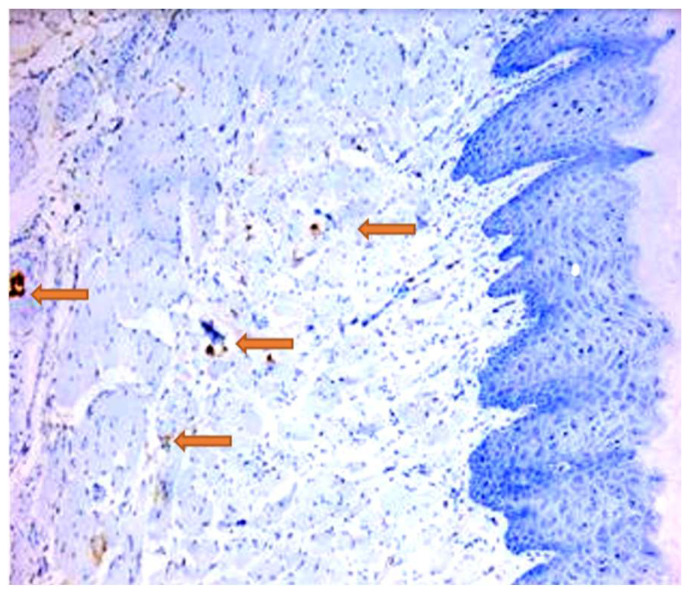
Rat, gingiva. Identification of vascular structures by CD34 immunolabelling; 100×.

Damage to the vascular wall in the form of microhemorrhages in the periodontal connective tissue was observed (Figure 6).

## 4. Discussion

Periodontal disease is characterized by a chronic bacterial infection that gives rise to inflammatory lesions and provokes a robust immune response. The immune response serves a dual role: it safeguards the host by controlling the infection, while simultaneously causing gradual damage to the supporting mechanisms of teeth within the alveoli [44]. This dual effect arises from the action of inflammatory mediators and immune cells. Among these immune cells, activated T and B lymphocytes play a pivotal role in both controlling periodontal infections and determining the extent of destruction in periodontal tissues [45].

Conversely, diabetes is associated with serious complications that stem from a chronic inflammatory environment. This includes inflammation of periodontal tissues and bone loss, driven by alterations in host metabolism resulting from hyperglycemia [44,46]. Diabetes induces changes in immune cell function, the release of pro-inflammatory cytokines from PMN and monocytes as well as a reduction in macrophage growth factors, mainly. These modifications predispose the body to chronic inflammation, progressive tissue degradation, and reduced repair capacity. Periodontal tissues are particularly susceptible as they are constantly exposed to endotoxins from bacterial biofilms [17]. All available evidence pointing to the biological connection between diabetes and periodontal disease indicates that diabetes and persistent hyperglycemia trigger an exaggerated immune-inflammatory response to periodontal pathogens, resulting in accelerated and severe destruction of periodontal tissues [47]. A growing body of research has demonstrated the occurrence of lesions in periodontal tissues in individuals with diabetes [2,4,48].

In our current research, we observed that both at the gingival epithelium and chorion levels, CD3+ T lymphocytes were the most abundant cell type, followed by CD20+ B lymphocytes, across all groups of rats studied. This observation contrasted with the control group, where although present, these inflammatory-type cells did not contribute to tissue destruction but rather played a role in the host’s response to bacteria and other substances encountered by the gum. Earlier studies have also highlighted that significant numbers of T and B lymphocytes infiltrate the gingival tissue in an antigen-specific manner [49,50,51]. Researchers have attributed this inflammatory response to bacterial products within plaque, which interact with the gingival epithelium, inducing the expression of adhesion molecules, proinflammatory cytokines, and chemokines. These molecules guide leukocytes into the gingival tissue and ultimately into the gingival sulcus through the junctional epithelium. Within the connective tissue, an inflammatory infiltrate predominantly composed of T lymphocytes is formed.

Subsequently, this adaptive response shifts the nature of the inflammatory response to involve B cells and plasma cells. This shift can lead either to the production of protective antibodies and the subsequent control of infection or the production of non-protective antibodies, contributing to connective tissue destruction and bone loss [52]. It has been proposed that T cells predominate in the initial stages of periodontal disease, with an increase in B cells in more advanced stages, albeit still under the regulation of T cells [12,52,53]. This may elucidate why CD20+ B lymphocytes were the second most prominent subgroup of immune cells affected in our study [54]. The exact mechanism is still unclear. It seems that B cells generate IL-10 and TGF-β as proinflammatory mediators and TNF-α, IL-6, and matrix metalloproteinases as anti-inflammatory factors [55].

At sites of inflammation, CD20+ B lymphocytes predominated in some sections, displaying either a diffuse distribution or clustering around capillaries, angiogenesis vessels, or forming nodular structures. These observations likely correlate with the intensity of the inflammatory process and the presence of antigens. The clustering around capillaries and angiogenesis vessels can be attributed to the transportation of antigens through the bloodstream to these areas [56].

Numerous investigations have unveiled diabetes’ contribution to periodontal disease and its severity, affecting both the inflammatory and immune responses as well as the vascular system [1]. Microvascular alterations represent one of the initial factors in the development of gingivitis and its progression to periodontal disease, potentially due to the transportation of pro-inflammatory cells and the increase in endothelial surface area, leading to the production of cytokines and other factors that drive inflammation forward [57]. In our study, the local levels of total vascular structures expressing CD34, mature blood vessels CD34+, and immature blood vessels CD34+ were not significantly altered in the DP group compared to the control group, possibly because the periodontal changes were not highly advanced. An association between an increased number of blood vessels and the progression of periodontal disease, characterized by pronounced thickening of basement membranes, particularly in capillaries and venules, has been reported [58]. The microhemorrhages observed in the periodontal connective tissue in our current research are likely the result of local mechanical trauma and vascular damage caused by the heightened aggressiveness of microorganisms in the bacterial plaque, either directly or through the production of toxic substances.

Recent scientific interest has surged in assessing the potential benefits of natural extracts in modulating the inflammatory and immune responses [59,60,61]. In our study, Cu, a highly potent polyphenolic compound derived from turmeric, specifically isolated from Curcuma longa, has exhibited anti-inflammatory and anti-proliferative properties. Cu demonstrated interactions with various immune cells, including dendritic cells, macrophages, natural killer cells, neutrophils, T and B lymphocytes [62]. Its immunomodulatory effects primarily involve immune system suppression [63]. With respect to T cells, Cu influences proliferation by downregulating activated cells or by blocking constitutively activated targets in T cell signaling pathways, thereby suppressing proliferation [64]. Additionally, Cu promotes regulatory T cell differentiation, induces T cell apoptosis, and inhibits T cell activation [65]. Our results converge with a previous study in which orally administered Cu inhibited inflammation through antioxidative stress mechanisms both locally and generally in periodontitis rats with diabetes [30]. Oxidative stress biomarkers (malondialdehyde) were reduced and antioxidants (glutathione, oxidized glutathione, glutathione/oxidized glutathione and catalase were increased following Cu administration.

In another induced periodontitis in a diabetic rat experiment, Cu reduced the infiltration of PMN and monocytes, the degradation of periodontal collagen fibers—and decreased IL-1β, IL-6, TNF-α MMP-9 [66] and MMP-2, MMP-8 levels [67]. Moreover, Cu exhibited hypoglycemic effects via an oxidative stress mechanism. Cu anti-inflammatory effects were increased while bone resorption was alleviated when a chemically modified curcumin, designed to counteract Cu’s lack of bioavailability, was used [66]. Later on, the same authors found the suppression of p38 MAPK and NF-κB signaling pathways to be modulated by the modified Cu [68]. Incorporated in nanoparticles, Cu decreased the periodontal tissues’ inflammatory infiltrate and also, the number of osteoclasts and reduced the activation of p38 MAPK and NF-kB [23].

Little evidence exists regarding potential therapeutic agents with modulatory effects on B cells. Although less studied in relation to B lymphocytes, Cu was found to suppress their proliferation and function and also modulate B cell differentiation [69]. Cu, as an anti-CD20, was investigated and it seems it can inhibit the activation, proliferation, and differentiation of naïve B cells into B effector cells. Cu, also impaired upon B cells function by inhibiting the release of auto-antibodies from B effector cells [69]. The modification of Cu on the CD+ 20 was not relevant in the present study. There is increased interest in using CD 20 as a target in B cell pathologies treatments, but the results are unclear due to an incomplete understanding in B cells metabolism [70].

Recently, there has been much interest in the immature CD34+ blood vessels or progenitor cells, that can circulate, proliferate, and differentiate into mature endothelial cells [71]. Progenitor endothelial cells circulate in the blood and seem to preferentially settle to vascular or tissue lesions, significantly contributing to both reendothelialization and neo angiogenesis and therefore playing a key role in maintaining vascular endothelial function [72]. The increase of immature CD34+ blood vessels in the Cu treated group (DPCu) could be a result of Cu stimulating the proliferation of the progenitor cells in a local regenerative process. Our observations are consistent with recent treatment directions that evaluate the effects of CD34+ cell therapy in various diseases [72]. Authors reported that the mechanism of local healing involved low release of pro-inflammatory agents IL-1, IL-6, TNF-α, and nitric oxide synthase 2 (an enzyme which is encoded by the reactive free radical nitric oxide), and overexpression of IL-10 in CD34+ cell therapy [72,73]. Our outcomes are similar to another other study where the authors advanced the idea of antagonistic effects of Cu depending on the dose, time, and type of cells that are targeted [74,75]. This could explain the lack of effect of Cu in the DPCuR group where the dose of Cu was halved compared to the one in DPCu group.

R, also known as P Vitamin, rutoside, quercetin-3-rutinoside, and sophorin, is a flavonoid widely present in plants that showed, among many others, anti-oxidative stress and anti-inflammation properties [76,77,78]. While there is limited direct evidence on the specific impact of R on T and B lymphocytes, several studies have identified R as an anti-inflammatory agent. It has been shown to reduce pro-inflammatory biomarkers such as tumor necrosis factor-α, interleukin (IL)-6, cyclooxygenase-2, and IL-1β [79]. These biomarkers are closely interconnected with T and B lymphocytes at various levels. They are either produced or expressed by these lymphocytes or can downregulate their suppressive activity [79,80,81,82,83,84]. R was recently studied in relation to the ligament periodontal cells. In an in vitro experiment, R augmented the antioxidant capacity and stimulated the proliferation of the human periodontal ligament stem cells [85] and osteogenic differentiation through the phosphatidylinositol 3-kinase/protein kinase B signaling pathway [28]. Another research study reported the mTOR signaling pathway being suppressed by R, shielding human periodontal ligament stem cells from TNF-α-induced damage to osteogenic development [85]. Several researchers proposed R as a therapeutic agent in advanced periodontitis [86]. R has been demonstrated to modulate the glucose and lipid metabolism, augment insulin levels, and have a protective role on pancreatic cells [76].

In our study, R, whether used alone or in combination with Cu, exhibited a significant reduction in the levels of T lymphocytes in the context of periodontal disease associated with diabetes. Moreover, this reduction was more pronounced when R was combined with Cu compared to Cu alone. Notably, in the combined treatment group, the levels of B lymphocytes were even more decreased than in the group treated solely with Cu. This suggests the possibility of a synergistic anti-inflammatory effect when Cu and R are used together. This research converges with results from a previous study in which gingival and circulatory oxidative stress was modulated by R in periodontitis diabetic rats. The effects were augmented by the R combination with C [30]. Malondialdehyde as a biomarker of oxidative stress was depleted and glutathione, oxidized glutathione, glutathione/oxidized glutathione and catalase, indicating the antioxidant response, were augmented. The fact that Cu associated with R had a more important anti-inflammatory effect is sustained by the results of other studies [87]. Several extracts have been found to exert superior antimicrobial and anti-inflammatory effects on periodontal disease when they were combined rather than when they were given separately [87].

Another two novel approaches, photobiomodulation [88] and probiotic therapy [89], could be tested in combination with Cu and R in order to understand their mutual effects on oral microbiota, and consecutively on periodontal disease onset and progression.

Developing the experiment using larger sample sizes and/or more concentrated treatments over a longer period of time could result in a more significant outcome.

Since Cu and R are known for their rapid metabolization when administered orally [90,91], in future research endeavors, it is crucial to explore the pharmacokinetics of Cu and R, with a particular emphasis on improving their solubility and bioavailability. Recent studies reported promising results when nanotechnology was used for developing new Cu and R delivery formulas [92,93,94].

## 5. Conclusions

The administration of Curcumin (Cu) and Rutin (R), whether individually or in combination, resulted in a reduction of the immunohistochemical markers CD3, CD20, and CD34 within the gingival tissues of Wistar rats afflicted with diabetes-associated periodontal disease.

## Figures and Tables

**Table 1:** Immunohistochemical markers used.

Antibody	Epitop/Marker	Manufacturer	Antigenic Unmasking	Dilution
CD3	T lymphocytes	DAKO	Citrate buffer pH = 6	1:100
CD20	B lymphocytes	DAKO	Citrate buffer pH = 6	1:50
CD34	Endothelial cells	DAKO	Citrate buffer pH = 6	1:50

**Table 2:** Mean Values of tested biomarkers.

Group	T CD3+ Cells	B CD20+ Cells	Total CD34+ Cells	Mature CD34+ Cells	Imature CD34+ Cells
Control	3	2.5	17.5	7	10.5
DP	55.5	6	13.5	4	9.5
DPCu	41	3.5	25	4.5	20.5
DPR	15	6.5	7	2.5	4.5
DPCuR	2	0.5	8	3	5

**Table 3:** Differences for CD3+ T and CD20+ B in groups.

Control Group	Mean	SD	t	*p* Value
T CD3+ cells	3	0.001	11.7611	˂0.0001
B CD20+ cells	2.35	0.231		
DP	Mean	SD	t	*p* value
T CD3+ cells	54.4	0.83	199.20	˂0.0001
B CD20+ cells	5.73	0.46		
DPCu	Mean	SD	t	*p* value
T CD3+ cells	40.6	0.50	275.09	˂0.0001
B CD20+ cells	3.4	0.13		
DPR	Mean	SD	t	*p* value
T CD3+ cells	14.4	0.5	59.2915	˂0.0001
B CD20+ cells	6.4	0.12		
DPCuR	Mean	SD	t	*p* value
T CD3+ cells	1.894	0.119	45.0619	˂0.0001
B CD20+ cells	0.49	0.012		

## Data Availability

The study did not report any data.
